# Healthy Diets in Rural Victoria—Cheaper than Unhealthy Alternatives, Yet Unaffordable

**DOI:** 10.3390/ijerph15112469

**Published:** 2018-11-05

**Authors:** Penelope Love, Jillian Whelan, Colin Bell, Felicity Grainger, Cherie Russell, Meron Lewis, Amanda Lee

**Affiliations:** 1 Institute for Physical Activity and Nutrition (IPAN), Deakin University, Geelong 3220, Australia; caru@deakin.edu.au; 2 School of Exercise and Nutrition Sciences, Deakin University, Geelong 3220, Australia; fgrainge@deakin.edu.au; 3 School of Medicine, Global Obesity Centre, Deakin University, Geelong 3220, Australia; jill.whelan@deakin.edu.au (J.W.); colin.bell@deakin.edu.au (C.B.); 4 The Australian Prevention Partnership Centre (TAPPC), Sax Institute, Sydney 2007, Australia; meron.lewis@saxinstitute.org.au (M.L.); amanda.lee@saxinstitute.org.au (A.L.); 5 School of Public Health, University of Queensland, Herston QLD 4006, Australia

**Keywords:** Healthy Diets ASAP tool, food security, food prices, diet affordability, rural communities, INFORMAS

## Abstract

Rural communities experience higher rates of obesity and reduced food security compared with urban communities. The perception that healthy foods are expensive contributes to poor dietary choices. Providing an accessible, available, affordable healthy food supply is an equitable way to improve the nutritional quality of the diet for a community, however, local food supply data are rarely available for small rural towns. This study used the Healthy Diets ASAP tool to assess price, price differential and affordability of recommended (healthy) and current diets in a rural Local Government Area (LGA) (pop ≈ 7000; 10 towns) in Victoria, Australia. All retail food outlets were surveyed (*n* = 40). The four most populous towns had supermarkets; remaining towns had one general store each. Seven towns had café/take-away outlets, and all towns had at least one hotel/pub. For all towns the current unhealthy diet was more expensive than the recommended healthy diet, with 59.5% of the current food budget spent on discretionary items. Affordability of the healthy diet accounted for 30–32% of disposable income. This study confirms that while a healthy diet is less expensive than the current unhealthier diet, affordability is a challenge for rural communities. Food security is reduced further with restricted geographical access, a limited healthy food supply, and higher food prices.

## 1. Introduction

*‘If it’s not available or you cannot afford it, then you cannot eat it even if you wanted to!’*.

[1] (p. 363)

The cost of food and the financial resources to procure it are key economic determinants of food choice [1]. Food security is defined as the physical, social and economic access to a stable and safe food supply, in sufficient quantity and quality to meet dietary needs and food preferences, within an environment that supports a healthy and active lifestyle [2]. In high income countries, like Australia, people identified as being most at risk for food insecurity have typically been those on low incomes, experiencing homelessness, refugees and migrants, and Aboriginal and Torres Strait Islander communities [3]. More recently, however, households on middle incomes, experiencing financial stress, have been identified as food insecure [3]. The national food insecurity prevalence of 4% [4] for Australia is therefore considered an underestimation, with predictions of 10–25% of households in some areas being food insecure [3].

The link between food insecurity and overweight/obesity [5] is of particular concern given the global prevalence of this complex public health problem [6]. In Australia, 25% of children and 63% of adults are overweight/obese [4], with rural Australian communities generally experiencing higher rates of obesity and decreased food security than their urban counterparts [7]. Despite having a higher disease burden, rural communities in Australia are frequently overlooked and under-researched regarding prevention, and therefore less informed about appropriate solutions. Providing an accessible, available and affordable healthy food supply is a well-established [8,9] and equitable way to improve the nutritional quality of food consumed by a community or population [10].

Unhealthy diets, and associated overweight/obesity, are now the major preventable risk factor contributing to the burden of disease [11], yet adherence to the Australian Dietary Guidelines is poor [12]. Unhealthy diets are caused by a range of complex and inter-related determinants including ‘obesogenic’ food environments, defined as an environment that promotes weight gain and hinders weight loss, affecting food promotion, availability, accessibility and affordability [9]. A key determinant is the perception that healthy diets are expensive and a barrier to the purchase of healthier foods [13]. Increased food prices, poorer quality produce and a limited variety of healthier options are primary contributors to food insecurity for Australian households [14]. The price and affordability of a healthy diet is of particular concern for rural communities where geographic location and low population density pose significant challenges for the food supply chain, resulting in an infrequent supply of healthy food to at risk communities [15], often of poorer quality [16] and less varied in terms of product brand, size and type [17,18]. A lack of infrastructure in these areas with low-density transport networks and high car dependency also make access to food outlets more difficult than in larger towns and cities [15].

Food pricing information in Australia has most commonly been collected using “healthy food basket” (HFB) methodology, using a predefined list of indicator food items representative of the total diet for different reference households [13]. Different HFB methodology exists across Australian States and Territories, with Victoria using the Victorian Healthy Food Basket (VHFB) comprising 44 listed food items. The VHFB approach poses limitations for small rural towns with food stores that often do not meet the inclusion criterion to stock at least 90% of the listed food items [19] as well as not including generic food product brands which are becoming increasingly prominent in Australian food stores [20].

The recent development of the Healthy Diets Australian Standardised Affordability and Price (ASAP) tool, through the global INFORMAS (International Network for Food and Obesity/non-communicable diseases Research, Monitoring and Action Support) network, may overcome these challenges. The Healthy Diets ASAP tool seeks to provide a standardised method to assess and compare the price and affordability of the recommended Australian diet with the current Australian diet [21,22,23], to enable informed community specific food supply decisions. The Healthy Diets ASAP tool comprises 76 food items [23] indicative of the recommended Australian diet (based on the quantitative modelled Foundation Diets within the Australian Dietary Guidelines) [24] and the current Australian diet (based on reported dietary intakes within the Australian Health Survey 2011–2012) [25]. Food item prices (*n* = 43), adjusted for edible proportion, representative of the recommended Australian diet encompass the five food groups (fruit; vegetables and legumes; grain/cereal foods; meats, poultry, fish and alternatives; milk, yoghurt, cheese and alternatives); and unsaturated oils and spreads. Additional food item prices (*n* = 33) representative of the current Australian diet include discretionary high in saturated fats, sugars, salt and/or alcohol (described as energy dense) and considered not necessary as part of a healthy diet. Discretionary items include cakes, biscuits, pastries, pies; chocolate, confectionary, ice confections; butter, cream, spreads which contain predominantly saturated fats; potato chips, crisps and other fatty or salty snack foods; sugar-sweetened soft drinks and cordials; sports and energy drinks; and alcoholic drinks) [24] (p. 144). 

This study assessed and compared the price, price differential (relative price) and affordability of the recommended Australian diet (as defined by the Australian Dietary Guidelines) and the current Australian diet (as described by the Australian Health Survey) for a small rural Local Government Area in Victoria, Australia. Ethical approval for this study was obtained through Deakin University (HEAG-H 80_2016).

## 2. Materials and Methods

### 2.1. Study Context and Selection of Study Site

The LGA selected for this study was determined by its rurality, modifiable chronic disease risk factor profile, and limited exposure to State-funded health promotion/obesity prevention initiatives.

The study LGA is predominately a rural area, growing mainly wheat, barley, oilseeds and legumes, and grazing sheep [26]. Geographically classified as remote (population size 5000–10,000), the LGA is described as having moderate accessibility based on minimum road distance from populated localities to nearest service centres [27]. At the time of this study, the LGA had a total population of 6674 residents across 7158 km^2^, comprising one main town (≈2300 residents), eight small towns (≈130 to 800 residents) and eight smaller localities (<100 residents) [26]. The LGA is subdivided into three wards (north, central, south) defined by electoral boundaries, influencing the provision of services and creating three distinct community hubs within the LGA. The LGA scores below the regional State average on the Index of Relative Disadvantage, with up to 59% of families in some towns on low incomes; a high proportion of people aged over 65 and people with a disability; and high levels of social isolation [28,29]. Compared with State averages, the LGA experiences a high prevalence of overweight (38.3% vs. Victorian average 31.2%) and obesity (38.3% vs. Victorian average 18.8%); low fruit and vegetable consumption (4.5% vs. Victorian average 5.2%); high sugar sweetened beverage consumption (30.3% vs. Victorian average 15.9%); high take-away meal consumption (80.9% eating takeaway once per week vs. Victorian average 71.2%); and similar levels of food insecurity (4.6%) [30].

### 2.2. Selection of Data Collection Tool

The Healthy Diets ASAP tool was used to collect food pricing information (Supplementary File S1) [21,23] for the recommended Australian diet, defined by the Australian Dietary Guidelines (ADG) [24], and the current Australian diet, described by the Australian Health Survey (AHS) [25] (Table 1). The current Australian diet is comparable with that reported for the study LGA [30]; namely; low daily fruit consumption (LGA 1.3 serves vs. AHS 1.2 serves vs. ADG 2 serves); low daily vegetable consumption (LGA 2.5 serves vs. AHS 2.7 serves vs. ADG 5 serves).

### 2.3. Selection of Retail Food Outlets

Thirty-nine retail food outlets (supermarkets, general stores, bakeries, take-away outlets, cafes, hotels/pubs and service stations) were identified across the LGA using the community directory available on the LGA website. Validation of these business listings, using ‘ground truthing’ (physically viewing and recording of outlets) [31], identified that three outlets had closed and four new outlets had opened. All outlets operating at the time of the study were surveyed (*n* = 40). These outlets were located across ten towns within the LGA.

### 2.4. Data Collection

Assistance was provided by AL and ML regarding the use of the Healthy Diets ASAP tool protocols [23] and data were collected by four researchers, working in pairs (PL, JW, FG, CR), within one week in June 2017. As per protocol, within each town, all supermarkets and general stores were surveyed first, followed by bakeries, take-away outlets, cafes, hotels/pubs, and service stations. Permission to participate was obtained verbally from each outlet manager immediately prior to data collection, with all outlets agreeing to participate. Data collected included usual price for specified brands and sizes; sale/special promotion price if usual price was unavailable; price of cheapest brand if specified brand was unavailable; price of nearest larger size (or nearest smaller size) if specified size was unavailable; and cheapest usual price for loose fresh produce. Alternate product brand names and sizes were recorded. Unavailable items were cross checked with outlet managers to determine if out of stock or never stocked. Information for out of stock items was provided by outlet managers, and never stocked items were recorded as missing.

### 2.5. Data Entry

Eleven data sheets were compiled representing the main town with two supermarkets, and the nine smaller towns each with one supermarket or general store. Data entry was done by F.G. and C.R. with all entries cross-checked by PL and JW. As per protocol, missing items within an outlet were allocated the mean price for that item from all other outlets across the LGA.; and price conversions were calculated for alternate product sizes. The Healthy Diets ASAP tool uses the reference household of two parents (one full-time employed; one part-time employed) and two children (boy aged 14 years; girl aged 8 years). Median disposable family income for this reference household was derived from recent census data for the LGA, calculated at $AUD2358/fortnight [26]. Using the Healthy Diets ASAP tool protocol, indicative minimum disposable income for this reference household was calculated based on minimum wage rates, family tax benefits and relevant welfare payments derived from the Australian Government Department of Human Services [32], calculated at $AUD2167.24/fortnight as detailed in Table 2. The LGA scores below the regional State average on the Index of Relative Disadvantage [28] with only 7.3% of households on high incomes [26].

### 2.6. Data Analysis

Data were analysed to explore price differential and affordability of the recommended diet and current diet for the reference household for the whole LGA; by ward (south, central and north); and each town within the LGA. Mean food prices were used for whole of LGA and by ward analyses. Price differentials were compared using the following metrics: total diet; each of the five food groups (fruit; vegetables/legumes; grains/cereals; meats/nuts/seeds/eggs; and milk/yoghurt/cheese); unsaturated oils/spreads; discretionary items (take-away foods, soft drinks, alcoholic beverages). Data were also entered into SPSS version 25 and Wilcoxon-signed ranks test were used to compare total diet costs between towns, and between the northern, central and southern areas of the LGA. Affordability of the recommended diet and current diet was calculated as a proportion of household income using median and indicative minimum disposable incomes for an average and low income household, respectively.

## 3. Results

**Figure 1:**
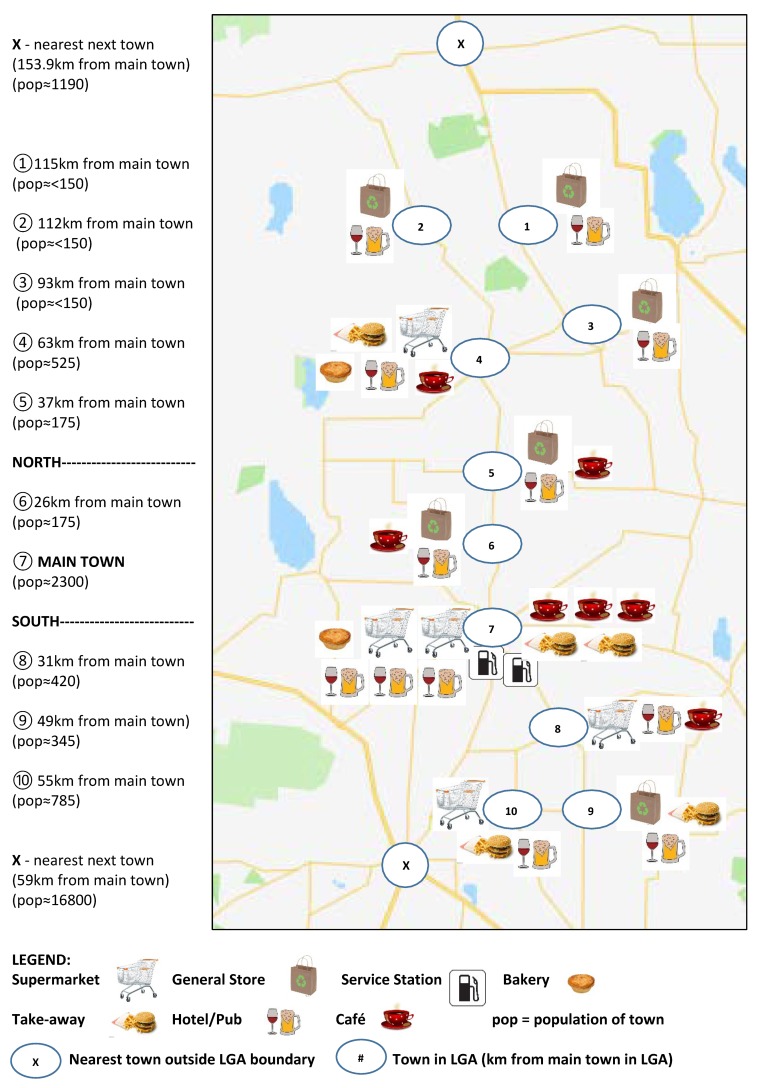
Distribution of food retail outlets across ten towns within the Local Government Area indicating type of outlet and distance (km) of towns from the main town (https://www.google.com/maps).

Forty retail food outlets were included in the study, located across 10 towns, and categorized as supermarkets (*n* = 5), general stores (*n* = 6), bakeries (*n* = 2), take-away outlets (*n* = 6), cafés (*n* = 7), hotels/pubs (*n* = 12) and service stations (*n* = 2) (Table 3). The majority of outlets (*n* = 14) were in the main town, with a range of 2–5 outlets in the smaller towns. Supermarkets were located in four towns; two in the main town and one each in the three next most populated towns. General stores were located in the six remaining towns. All towns had at least one hotel/pub, and the majority of towns had a café and/or take-away outlet. Three towns, all with populations less than 150, had no cafés or take-away outlets (Figure 1).

**Figure 2:**
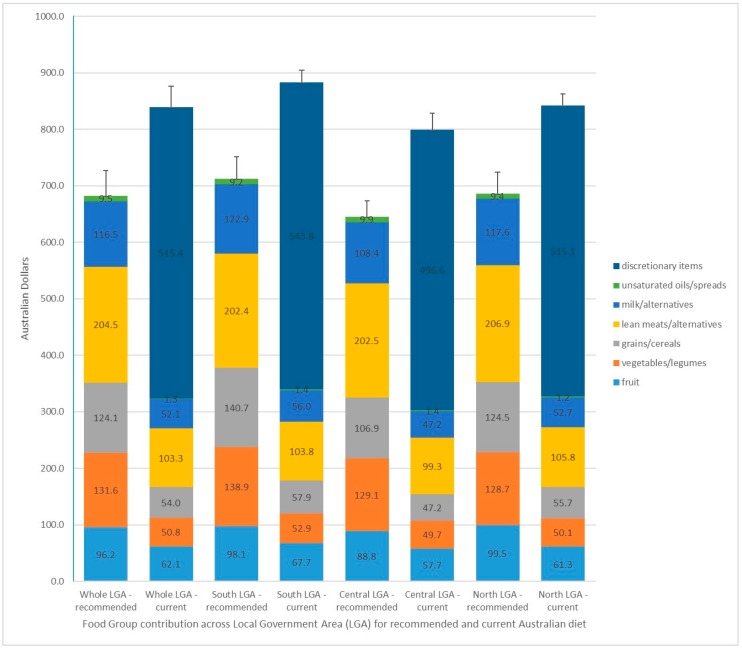
Food group contribution and total diet costs for recommended and current diets for the Local Government Area ($AUDmean ± SD per fortnight).

Pricing for the recommended and current diets, using the reference household, are presented for the whole LGA, and the southern, central and northern communities of the LGA in Figure 2 and Appendix A. Figure 2 also illustrates the contribution of the cost of component food groups to total diet costs. Data for each town is available in Supplementary File S2.

Across the LGA, the recommended diet was cheaper than the current diet ($AUD702.41 ± 44.80 vs. $AUD866.19 ± 37.54 per fortnight/reference household), costing an average 81.1% of the current diet budget. Within the current diet, expenditure for all five food groups was less than half what would be required to achieve recommended intakes of these foods; namely, fruit and vegetables (13% current vs. 32% required); grains/cereal-based foods (6.2% current vs. 17.7% required); lean meats, poultry, fish, eggs (11.9% current vs. 29.1% required); and milk, cheese, yoghurt (6.0% vs. 16.6%). The majority of the current diet budget was spent on discretionary items (59.5%), particularly take-away foods/beverages (18.1%) and alcoholic beverages (11.2%).

For each of the three LGA wards, the recommended diet was cheaper than the current diet, costing an average 81.3% of the current diet budget for southern and northern wards, and an average of 80.4% for central towns (Supplementary File S2). Food item prices were higher in the southern (8.7%) and northern (5.5%) towns than the central towns in the LGA. Food item prices in the southern towns were highest for four of the five food groups (especially fruits, grains, and milk, cheese and yoghurt); take-away food items were the most expensive (approximately 12–13%); and sugar-sweetened beverages were the cheapest. For towns in the centre of the LGA, take-away food items were the cheapest and sugar-sweetened beverages the most expensive (approximately 19–23%). For both the southern and northern towns, price differences were greatest for grains (approximately 15–19%) and milk, cheese and yoghurt (approximately 10–16%) than the other five food groups.

Across the LGA the cost of the current diet was statistically significantly higher than the recommended diet at *p* < 0.05. There were no significant differences between the three LGA wards.

Affordability of the recommended and current diets was calculated using median and indicative minimum disposable incomes for an average and low income household, respectively (Table 4). Across the LGA, the recommended diet ($AUD702.41 ± 44.80/fortnight/reference household) would expend 30–32% of a median and low income household, respectively; and the current diet ($AUD866.19 ± 37.54/fortnight/reference household) would expend 37–40% of a median and low income household. Affordability of the recommended diet as a proportion of household income was similar for the southern and northern towns, approximately 2% higher than towns in the centre of the LGA.

Availability of food items listed on the Healthy Diets ASAP tool protocol (*n* = 76) varied across the ten towns within the LGA. All items were available within the main town, which had two supermarkets and twelve other food retail outlets. Towns with supermarkets appeared to have fewer missing food items (3–12 missing items) compared to towns with only a general store (18–38 missing items). Most commonly missing food items were low fat yoghurt and low fat cheese (available only in the main town); cooked whole chicken (available in two towns); canned sweetcorn (no added salt) and extra virgin olive oil (both available in three towns); and unsalted peanuts (available in four towns). Yoghurt (full or reduced fat) was unavailable in five towns. Specific product brands and product sizes were unavailable for 36 (47%) and 43 (75%) of the listed food items, respectively, requiring substitution with price of cheapest brand and price of nearest larger (or nearest smaller) size. As per protocol, missing items within an outlet were allocated the mean price for that item from all other outlets across the LGA, thereby minimizing effects to food budget calculations.

## 4. Discussion

This study assessed the price, price differential (relative price) and affordability of the recommended and current diets in a small rural LGA in Victoria, Australia. Study findings confirm the paradoxical co-existence of food insecurity, low income and obesity, linked to limited geographical access to a healthy, fresh food supply; limited variety of healthy food options; the ubiquitous availability of highly palatable energy-dense foods, drinks and discretionary items; higher overall food prices; and low incomes reducing affordability of a healthy diet.

Findings suggest that a healthy diet, consistent with national dietary guidelines, is less expensive than the current diet consumed by Australians. For some rural towns, this differential may be as much as 18.9%. The Healthy Diets ASAP tool has only recently been used within Australia, with available studies of the pilot approach limited to urban areas reporting a finding of 16.3% for a low income household [21]. These findings challenge the perception that a healthy diet is more expensive, as described in a systematic review of food pricing studies across 10 countries (excluding Australia) [33] which found little difference between the cost of healthier and unhealthy dietary patterns. As explained by Lewis and Lee [13], studies included in this review did not consider the contribution of alcoholic beverages or most other discretionary items^1^ to the cost of the diet nor the application of a goods and sales tax (GST) exemption to certain food items. The inclusion of discretionary items is important in the Australian context, as over a third (35%) of the total daily intake of Australians comprises discretionary items in the form of biscuits, cakes, confectionary, sugar-sweetened beverages and alcoholic beverages [12]. Additionally Australia applies a 10% GST exemption to basic, healthy foods in the five food groups such as fruit, vegetables, bread, fresh meat, eggs and milk [34], increasing the affordability of these food items.

On average the income of Australian families in rural and remote areas is 15–20% lower than in metropolitan areas, which together with higher food prices in these areas, makes it difficult to afford a healthy diet [15]. While purchasing a recommended diet may be less expensive than the current diet, findings from this study highlight that it would account for almost a third of the budget for median (30%) and low (32%) income households in the LGA. These levels of affordability align with those reported by the Healthy Diets ASAP pilot tool, where a recommended diet accounted for 20–29% of a low income household budget in an urban area [21]. Study findings are similar however to research using ‘healthy food basket’ methodologies which found the recommended diet accounted for between 26–32% of a low income household budget across urban and rural towns of South Australia [17], Victoria [16,35] and Queensland [36]. While there is no accepted benchmark for affordability of a healthy diet [22], relative unaffordability is commonly associated with food costs accounting for 30% or more of the household budget [1,35]. Recently, Ward et al. [17] have proposed that ‘food stress’ occurs when food costs account for 25% or more of the household income.

Australian studies consistently show significant increases in food pricing as one moves from inner city to suburban to regional and rural areas [16,17,35,36]. “Out-shopping”, purchasing food outside of one’s local area from a larger centre, is therefore a common practice in rural areas [37] to benefit from lower prices and greater variety. In this study, differences in food prices were observed across the LGA, with prices 8–10% higher in the south. Furthermore, a comparison of the cost of a ‘Victorian Healthy Food Basket’ for the LGA ($AUD528.41), with neighbouring regional towns ($AUD438.30—town population ≈ 17,000; $AUD453.34—town population ≈ 30,000) reveals that food prices are lower in regional towns outside the LGA boundary [38,39]. In addition to higher food pricing and out-shopping, rural communities often experience low density transport networks, leading to an increased reliance on motor vehicles, with associated time, fuel and vehicle maintenance costs, when purchasing food [15].

This study also found that the majority of the food budget was spent on discretionary items (59.5%), of which take-away foods comprised 18%. The Australian Dietary Guidelines food price indexes report [40] estimates that 58.2% of the 2014 household food budget was spent on discretionary items; and Lee et al. [21] report a similar figure of 58% for households in an urban area. While study findings on discretionary item expenditure are higher than reported by others, they do align with the Victorian Population Health Survey [30], with the LGA having high sugar sweetened beverage consumption (30.3% vs. Victorian average 15.9%), low fruit and vegetable consumption (4.5% vs. Victorian average 5.2%) [30]; and high take-away meal consumption (80.9% eating takeaway once per week vs. Victorian average 71.2%).

While a recommended diet may cost less than the current diet, it would appear that price is not the main driver of food choice for this community. In addition to the cost of foods, LGA residents have reported poor quality, limited variety (especially culturally appropriate foods), and inadequate or unreliable public transport as other reasons limiting their food choices [28]. The abundant supply of ‘convenience’ outlets (bakeries, take-away outlets, cafes, hotels/pubs, service stations) also appears to meet consumer demand for convenience [32] and taste [14], overcoming the challenges of limited geographic access, busy lifestyles and limited cooking skills, while also contributing to the local economy.

The rising cost of foods [16] combined with a limited number of food retail outlets, stocking a reduced variety of food items, lowers the likelihood of rural communities adhering to a healthier diet [41]. Rural areas with few supermarkets and several ‘convenience’ outlets have been found to have higher food prices, and limited availability of fresh produce and healthier food choices, particularly skim/low fat milk, whole wheat bread, fruits and vegetables [42]. Of the 40 food retail outlets included in this study, 11 (27.5%) were supermarkets and general stores selling predominantly healthy five food group items; and 29 (72.5%) were ‘convenience’ outlets selling predominately discretionary and take-away food items. Rural towns in Victoria have consistently been excluded from ‘Victorian Healthy Food Basket’ studies as they do not meet the inclusion criterion of stocking at least 90% (40 of 44) of listed food items [16,19]. Similarly, this study found that healthier food items were frequently unavailable in smaller general stores compared with supermarkets.

There is potential for supermarkets to increase variety and quality of fresh and healthy food options; provide competitive, lower pricing for healthy foods; and improve geographic access in areas described as ‘food deserts’ [41]. However, studies in the United States show that while supermarkets improve the perceptions of healthy food access amongst residents, improvements in net availability of healthy foods may be minimal, with residents continuing to shop outside their local area; more food stores stocking a wider variety of all food products; and greater market segmentation with ‘convenience’ outlets reducing stocks of healthy foods [41]. Positive impacts on food pricing however may be experienced with healthy foods offered at lower prices and discretionary item prices increasing as ‘convenience’ outlets attempt to compensate for reduced stocks of healthy foods [41]. In Australia, supermarkets are described by Pulker et al. [43] (p. 1) as having “*a powerful position in the Australian food system…… acting as gate-keepers between food producers and consumers*”, thereby influencing the range and price of food choices available, and shaping consumer preferences and social norms. While Australian supermarkets demonstrate some commitment to nutrition promotion and the prevention of obesity, Sacks et al. [44] argue that more is needed across this sector, especially to address the availability, affordability and promotion of healthy food choices.

In contrast to the establishment of new supermarkets, improvements to existing stores is suggested as a less time consuming and less expensive strategy to improve the variety and relative price of healthy food options in underserved areas with ‘food deserts’ [41]. In-store activities found to be feasible and acceptable to food retailers in rural communities, with modest levels of effectiveness, appear to focus predominately on health promotional practices, such as the provision of recipes and shopping lists for healthy meals, in-store displays with healthy samples, promotional signage within the store, and point-of-purchase signage for fruits and vegetables [45]. In a recent systematic review of 30 studies across nine countries regarding the effectiveness of food pricing strategies, Gittelsohn et al. [46] found that nearly all studies (*n* = 27) used in-store pricing strategies to promote healthy foods, most commonly fruits and vegetables (usually through price discounts, coupons and vouchers). Few studies (*n* = 6) used pricing strategies to specifically discourage unhealthy foods such as sugar-sweetened beverages and foods high in fat and/or sugar (using a price increase). It was noted that using pricing strategies that target only fruits and vegetables may be difficult for small retail outlets to implement, especially in low income communities, as fresh produce is often hard to source and highly perishable; and therefore any price incentives should cover a broad range of healthy food items [46].

In one of few studies exploring the perspectives of retailers, Kim et al. [47] found that small store owners in a low income community, regardless of their ethnic background, regarded customer preferences and wholesaler availability of food products as critical barriers to the provision of healthy options. The stocking of ‘low customer demand’ items was perceived to be a high-risk investment resulting in possible sales loss. When queried about pricing strategies, concerns were raised about offering discounts on multiple items given the small range of products the stores usually stocked. Retailers felt that discounts created price fluctuations and customer dissatisfaction when prices returned to normal. They also described the availability and pricing/discounting of items as being highly dependent on what wholesalers can offer [47].

Rural communities in Australia are serviced by long food supply chains which are not flexible to sudden changes or able to keep inventories to a minimum; instead they encourage the delivery of set quotas of items with a long shelf life [15]. Small stores not aligned to major supermarket chains are therefore at a disadvantage in acquiring fresh produce regularly and at competitive prices. Strategies to improve and/or subsidise the freighting of food to remote Australian communities have been suggested for communities who face similar challenges of vast distances, extreme temperatures and variable road conditions [48,49]. For example, ‘group freight buying’ where a group of stores combines their volumes to fill transport units on a geographically logical freight route that are not at full capacity, resulting in increased service frequency and/or lower freight costs per unit transported [49]. Such strategies will require leadership across all levels of government, and a strong commitment to the development and implementation of a National Nutrition Policy [50] and a National Food Plan that considers health [51].

At a policy level, food pricing options exist in the form of taxation, subsidisation, or a combination of these [22,52]. The taxing of unhealthy foods is considered of benefit for raising revenue as well as an effective strategy to improve dietary behaviours [53]; and subsidising healthy food is considered of benefit in making these foods more affordable and also, though to a smaller effect, appear to improve dietary behaviours [21,52]. For Australia, the exemption of ‘healthy’ foods from goods and sales tax (GST) is a means of reducing ‘food stress’ for low income families. Without this safeguard, Lee et al. [21] estimate that the cost of a healthy (recommended) diet would increase by approximately 10%, with the likelihood of a greater proportion of the food budget being spent on discretionary items.

## 5. Strengths and Limitations

To our knowledge, this is the first study in rural Australia to utilize the Healthy Diets ASAP tool to explore the price, price differential and affordability of the recommended and current diet. This study was also able to survey all supermarkets and general stores given the relative low number of outlets available, thereby providing a true representation for this LGA. While an advantage to data collection, a small sample size of retail outlets poses limitations for statistically analysis.

As a cross sectional study, data collection reflects a single time point, occurring on random days of the week during Winter, and therefore pricing information is indicative of seasonal and wholesaler availability at that time. ‘Ground truthing’ was used to identify and verify the presence of operational food stores, however food environments are constantly subject to change, and food stores included in this study may have since closed and/or new businesses opened.

No data was collected regarding consumer shopping venue preferences, especially the phenomena of ‘out-shopping’ which is known to occur anecdotally; nor other means through which food items may be obtained such as the community garden, food swaps, the food pantry or food bank. It was also out of scope for this study to conduct in-depth interviews with food store owners which may have elicited information regarding food pricing strategies.

The Healthy Diets ASAP tool was a practical and time efficient survey to conduct across the LGA. While some product brands/sizes were different to those specified on the protocol, the tool allowed for alternate brands/sizes to be included. The use of average prices for missing/unavailable items may have led to an underestimation of the cost of the diet for these towns as residents would have travelled to purchase this item elsewhere. Information on unavailable items will be used to update the tool for greater utility. The tool has been developed for different reference households, with the default being two adults and two children. It may be necessary to enable a wider application to other reference households to better reflect the demographics of rural, remote communities with higher numbers of elderly couples with no children and single-parent families.

## 6. Conclusions

This study confirms that while a healthy diet is less expensive than the current unhealthy diet, affordability is a challenge for Australians living in rural Victoria, especially for families on median or low incomes. For these communities, food security is compromised by limited geographical access to food retail outlets, with most outlets, especially in smaller towns, offering a reduced variety of healthy food choices at higher prices.

Implications for research: Research shows that rural, remote communities have poor adherence to recommended dietary guidelines, experience higher rates of overweight/obesity and associated chronic disease, and are disproportionately affected by the influence of their food environment compared with their urban counterparts. There appears to be a gap, however, in research regarding the influence of food environments among rural communities. Continued research in this area is therefore warranted to improve our understanding and identification of important determinants of diet for these Australian communities.

Implications for practice and policy: It would appear that price is not the main driver of food choice for rural, remote Australian communities. A preference for unhealthier foods, that meet the needs of convenience and taste, undermines the establishment of a reliable demand-supply cycle that would be economically viable for small food retailers. The challenge of food distribution across vast distances to provide affordable, quality produce also serves as a barrier within rural communities affecting accessibility and availability of supply. Understanding the associations between these factors will help to shape appropriate interventions needed at the individual, organizational, community and policy level. It is evident that a combination of strategies is required, including public health campaigns and programs targeting the individual to improve food literacy knowledge and skills; interventions in food retail outlets to improve affordability and promotion of healthier foods/drinks; establishing alternative community-led food supply options such as food cooperatives, farmers’ markets and community gardens; safeguarding agricultural land use and monitoring the zoning of fast food retail outlets through local, regional and state government planning mechanisms; developing a flexible, responsive food supply chain; and retaining a General Sales Tax (GST) exemption for basic healthy foods.

## Figures and Tables

**Table 1:** Comparison of the recommended and current Australian diets for males and females (19–50 years) [12].

Food Groupings (Recommended Serves/Day)	Australian Dietary Guidelines—Recommended Dietary Intakes		Australian Health Survey—Current Dietary Intakes	
	Males	Females	Males	Females
Bread and Cereals	6	6	5.2	3.7
Fruit	2	2	1.2	1.1
Vegetables	6	5	2.8	2.7
Dairy	2.5	2.5	1.6	1.3
Meat and alternatives	3	2.5	2.2	1.6
Discretionary items	0	0	6.4	4.2

**Table 2:** Low income household calculations ($AUD) for reference household of two parents with two children within the Local Government Area (adult male; adult female; boy 14 years; girl 8 years).

Assumptions ^a^	Fortnightly Income	
The family is privately renting a 3 bedroom house at $130/week	Paid employment—adult male	$1390.04
The adult male works on a permanent basis at national minimum wage * ($18.29/h) for 38 h/week	Paid employment—adult female	$219.00
The adult female works on a part-time basis at national minimum wage * ($18.29/h) for 6 h/week	Family Tax Benefit A ^	$420.70
Both children attend school and are fully immunised	Family Tax Benefit A supplement	$55.87
None of the family are disabled	Family Tax Benefit B ^^	$108.64
The family have some emergency savings that earn negligible interest	Family Tax Benefit B Supplement	$13.62
The family has negligible tax deductions	Clean Energy Supplement	$9.94
	Rent Assistance **	$132.61
	INCOME TAX PAID #	−$185.66
	**TOTAL FORTNIGHTLY INCOME**	**$2167.24**

^a^ Verification of assumptions: https://profile.id.com.au/; * Minimum Wage: https://www.fairwork.gov.au/how-we-will-help/templates-and-guides/fact-sheets/minimum-workplace-entitlements/minimum-wages. # current-national-minimum-wage; ^ Family Tax A: https://www.humanservices.gov.au/customer/enablers/payment-rates-family-tax-benefit-part; ^^ Family Tax B: https://www.humanservices.gov.au/customer/enablers/payment-rates-family-tax-benefit-part-b; ** Rent Assistance: Full amount of rent assistance paid to couple with 1 or 2 children if rent is >$436.19 per fortnight, minimum rent is $229.8/fortnight. Full amount is $155.26/fortnight. Rent assistance is paid at the rate of 75 cents for every dollar of rent paid in excess of that threshold up to the maximum rate applicable to the person. Rental at $260/fortnight; rent assistance = 155.26 − [260 − 229.80 × 0.75] = $132.61 https://www.humanservices.gov.au/individuals/enablers/how-much-rent-assistance-you-can-get; # Income tax paid: (income tax due + income tax offset + remote area tax offset) => −5147.45 + 372.29 + 0 = $4775.16; Annual income tax due: tax bracket >$37,000 − $87,000 => $3572 plus 32.5c for each $1 over $37,000; Annual income at $41,847.52; Tax paid = 3572 + [(41,847.52 – 37,000.00) × 0.325] = $5147.45; Annual income tax offset: available if taxable income is <$66,667. Maximum tax offset of $445 applies if taxable income is $37,000 or less. This amount is reduced by 1.5 cents for each dollar over $37,000. Annual income at $41847.52; Tax offset = 445 − [(41,847.52 – 37,000.00) × 0.015] = $372.29 https://www.ato.gov.au/individuals/income-and-deductions/offsets-and-rebates/low-income-earners/.

**Table 3:** Number and type of retail food outlet surveyed across the Local Government Area (LGA).

Retail Food Outlet by Town		Super-Market ^a^	General Store ^b^	Bakery	Take-Away	Café	Hotel/Pub	Service Station	Total Outlets by Town
North of LGA	Town 1		1				1		2
	Town 2		1				1		2
	Town 3		1				1		2
	Town 4	1		1	1	1	1		5
	Town 5		1			1	1		3
Centre of LGA	Town 6		1			1	1		3
	Town 7	2		1	3	3	3	2	14
South of LGA	Town 8	1				1	1		3
	Town 9		1		1		1		3
	Town 10	1			1		1		3
Total Outlets by Type		5	6	2	6	7	12	2	40

^a^ Supermarket—chain store, selling food products predominantly, open for extended hours on most day; ^b^ General stores—privately owned, selling food products and other items, open for limited hours.

**Table 4:** Affordability (% household income) of the recommended diet and current diet across the Local Government Area (LGA) for the reference household by median and low income ($AUD).

LGA Area and Town	Median Household Income ($2358)		Low Household Income ($2167)	
	Recommended Diet (%)	Current Diet (%)	Recommended Diet (%)	Current Diet (%)
Whole of LGA	30	37	32	40
Town 1	28	36	30	39
Town 2	28	37	31	40
Town 3	32	37	35	41
Town 4	30	32	33	34
Town 5	32	38	34	42
North of the LGA	30	37	33	40
Town 6	27	35	30	38
Town 7	27–30	33–36	30–32	36–40
Centre of the LGA	28	35	31	38
Town 8	31	37	33	40
Town 9	33	39	36	42
Town 10	29	39	32	42
South of the LGA	30	37	32	40

## Supplementary Materials

The following are available online at http://www.mdpi.com/1660-4601/15/11/2469/s1, File S1: Healthy Diets ASAP tool collection sheet; File S2: Food pricing data for LGA and towns.

## Appendix A

**Table A1:** Price of recommended and current diets for the Local Government Area (using a reference household of two parents and two children).

Northern LGA = towns 1,2,3,4,5; Central LGA = towns 6,7; Southern LGA = towns 8,9,10	Whole LGA—Recommended Diet	Whole LGA—Current Diet	Southern LGA—Recommended Diet	Southern LGA—Current Diet	Central LGA—Recommended Diet	Central LGA—Current Diet	Northern LGA—Recommended Diet	Northern LGA—Current Diet
**TOTAL DIET $mean ±sd**	702.41 ± 44.89	866.19 ± 37.54	733.31 ± 39.70	901.38 ± 20.87	661.96 ± 27.66	823.23 ± 29.55	708.14 ± 37.96	870.86 ± 21.02
**CORE 5 FOOD GROUP FOODS** **$mean +sd (%total diet cost)**	702.41 ± 44.89 (100.0%)	343.47 ± 26.93 (39.65%)	733.31 ± 39.70 (100%)	360.24 ± 23.33 (39.97%)	661.96 ± 27.66 (100%)	318.79 ± 19.23 (38.72%)	708.14 ± 37.96(100%)	348.20 ± 22.15 (39.98%)
**FRUIT** **$mean ±sd (%total diet cost)**	96.20 ± 13.89 (13.70%)	62.06 ± 11.45 (7.16%)	98.14 ± 15.29 (13.38%)	67.73 ± 12.11 (7.51%)	88.77 ± 2.48 (13.41%)	57.65 ± 2.35 (7.00%)	99.49 ± 15.30 (14.05%)	61.31 ± 12.87 (7.04%)
**VEGETABLES/LEGUMES** **$mean ±sd (%total diet cost)**	131.61 ± 8.71 (18.74%)	50.77 ± 4.39 (5.86%)	138.87 ± 1.15 (18.94%)	52.90 ± 2.75 (5.87%)	129.14 ± 10.15 (19.51%)	49.74 ± 5.13 (6.04%)	128.74 ± 7.80 (18.18%)	50.10 ± 4.26 (5.75%)
**FRUIT & VEG/LEGUMES** **$mean ±sd (%total diet cost)**	227.81 ± 18.81 (32.43%)	112.83 ± 13.00 (13.03%)	237.01 ± 15.77 (32.32%)	120.64 ± 12.67 (13.38%)	217.90 ± 11.33 (32.92%)	107.39 ± 7.33 (13.04%)	228.23 ± 21.02 (32.23%)	111.41 ± 13.66 (12.79%)
**GRAINS/CEREALS** **$mean ±sd (%total diet cost)**	124.11 ± 17.69 (17.67%)	53.98 ± 5.13 (6.23%)	140.70 ± 13.93 (19.19%)	57.93 ± 3.86 (6.43%)	106.86 ± 8.71 (16.14%)	47.22 ± 2.06 (5.74%)	124.50 ± 13.52(17.58%)	55.68 ± 2.63 (6.39%)
**LEAN MEATS & ALT $mean ±sd (%total diet cost)**	204.48 ± 9.05 (29.11%)	103.27 ± 6.56 (11.92%)	202.41 ± 11.03 (27.60%)	103.80 ± 6.06 (11.44%)	202.54 ± 10.10 (30.60%)	99.32 ± 7.22 (12.06%)	206.88 ± 5.94 (29.22%)	105.76 ± 5.08 (12.14%)
**MILK & ALT** **$mean ±sd (%total diet cost)**	116.54 ± 6.64 (16.59%)	52.08 ± 4.82 (6.01%)	122.86 ± 3.30 (16.75%)	55.99 ± 4.79 (6.21%)	108.40 ± 5.41 (16.38%)	47.17 ± 2.80 (5.73%)	117.64 ± 2.83 (16.61%)	52.67 ± 2.94 (6.05%)
**UNSATURATED OILS/SPREADS $mean +sd (%total diet cost)**	9.47 ± 0.86 (1.35%)	1.30 ± 0.19 (1.15%)	9.16 ± 1.25 (1.25%)	1.43 ± 0.19 (0.16%)	9.92 ± 0.22 (1.50%)	1.36 ± 0.07 (0.17%)	9.39 ± 0.69 (1.33%)	1.19 ± 0.17 (0.14%)
**WATER$mean +sd (%total diet cost)**	20.00 ± 5.90 (2.85%)	20.00 ± 5.90 (2.31%)	21.18 ± 8.31 (2.89%)	21.18 ± 8.31 (2.35%)	16.33 ± 5.55 (2.47%)	16.33 ± 5.55 (1.98%)	21.50 ± 2.32 (3.04%)	21.50 ± 2.32 (2.47%)
**ALL DISCRETIONARY FOODS** **$mean +sd (%total diet cost)**		515.42 ± 22.77 (59.50%)		534.77 ± 28.41 (59.33%)		496.63 ± 17.77 (60.33%)		515.08 ± 5.49 (59.15%)
**TAKE-AWAY FOODS $mean +sd (%total diet cost)**		156.67 ± 15.29 (18.09%)		172.48 ± 19.10 (19.13%)		149.97 ± 3.06 (18.22%)		151.20 ± 9.14 (17.36%)
**ARTIFICIALLY SWEETENED BEVERAGES** **$mean +sd (%total diet cost)**		7.31 ± 1.10 (0.84%)		6.37 ± 0.93 (0.71%)		7.81 ± 0.91 (0.95%)		7.58 ± 0.96 (0.87%)
**SUGAR SWEETENED BEVERAGES$mean +sd (%total diet cost)**		44.03 ± 6.66 (5.08%)		38.37 ± 5.63 (4.26%)		47.05 ± 5.46(5.72%)		45.61 ± 5.79 (5.24%)
**ALCOHOLIC BEVERAGES** **$mean +sd (%total diet cost)**		97.33 ± 9.74 (11.24%)		102.63 ± 12.40 (11.39%)		88.45 ± 6.96 (10.74%)		99.47 ± 4.30 (11.42%)
