# Supplementary material for: Healthy Diets in Rural Victoria—Cheaper than Unhealthy Alternatives, Yet Unaffordable
Source: Int J Environ Res Public Health. 2018 Nov 5;15(11):2469. doi: 10.3390/ijerph15112469 (PMC6266685; doi:10.3390/ijerph15112469)
Supplement: Supplementary file 1 [file ijerph-15-02469-s001.zip › Supplementary File S1 - Healthy Diets ASAP tool collection sheet.pdf]

Healthy Diets ASAP (Australian Standardised Affordability and Price) Survey Form Final

Store name ________________________ Store Location: ________________ Date: ____________ Collector:___

NOTE: Please read the methods for collection on Page 2, prior to collecting data.

**Food**	**Specific brand**	**Your brand**	**Specific size**	**Your size**	**Your cost**	**Comments**
Bottled water, still	Mt Franklin		600mL			
Fruit						
Apples, red, loose			per kg			
Bananas, cavendish, loose			per kg			
Orange, loose			per kg			
Vegetables & Legumes						
White potato, loose, brushed/washed			per kg			
Tinned sweet corn, kernels, no added salt	Edgell		420g			
Broccoli, loose			per kg			
Cabbage, white, ½ cabbage (1/2=1.5kg) (weigh if necessary)			1.5kg			
Lettuce, iceberg, whole (1=0.6kg)			0.6kg			
Carrot, loose			per kg			
Pumpkin, ½ pumpkin (1/2 av. Jap=1.5kg, 1/2 av. Butternut=1kg) (weigh if necessary)			per kg			
Tinned 4 bean mix	Edgell		420g			
Tinned diced/chopped tomatoes, in tomato juice	Ardmona		400g			
Brown onion, loose			per kg			
Tomato, loose (not vine-ripened)			per kg			
Frozen mixed vegetables (cheapest specified brand)	Heinz, Birdseye or McCain		500g			
Frozen peas (cheapest specified brand)	Edgell, Birdseye or McCain		500g			
Tinned baked beans, in tomato sauce	Heinz		420g			
Grain (Cereal) Foods						
Weet-bix	Sanitarium		375g			
Wholemeal Bread	Tip Top Sunblest		650g			
Rolled oats, whole, Traditional (not quick oats)	Uncle Toby’s		1kg			
White Bread	Tip Top Sunblest		650g			
Cornflakes	Kellogg’s		725g			
Spaghetti (white)	San Remo		500g			
White rice, medium grain	SunRice		1kg			
Water Crackers, plain	Arnott’s		125g			
Meats, Poultry, Fish & Alternatives						
Lean beef mince (not heart smart)	Pre-pack(not vacuum)		per kg			
Lamb loin chops	Pre-pack		per kg			
Beef rump steak	Pre-pack		per kg			
Tuna, canned in vegetable oil, unflavoured (cheapest specified brand)	John West, Greenseas or Sirena		185g			
Whole Barbeque Chicken, cooked - Large/ Family	Supermarket		Per unit ~1.5kg			
Eggs, dozen, Free Range	Sunnyqueen Farms		700g			
Milk, Yoghurt, Cheese & Alternatives						
Cheddar cheese, regular fat	Coon		250g			
Cheddar cheese, reduced fat	Coon		250g			
Full cream milk, fresh	Paul’s or Dairy Farmers		2L			
Reduced fat milk, fresh (not skim)	Paul’s Trim or Dairy Farmers Lite		2L			
Plain Yoghurt, natural, Greek, regular fat (~4% fat)	Jalna		1kg			
Yoghurt, vanilla/flavoured, reduced fat (~1% fat)	Jalna		1kg			
Unsaturated Oils & Spreads						
Canola Margarine, regular fat	MeadowLea		500g			
Sunflower oil	Crisco		750mL			
Olive oil, Traditional (not extra virgin)	Moro		1 Litre			
Other – core foods not in both baskets & mixed foods						
Pre-made Chicken & Salad Sandwich (wholemeal) (1 sandwich = ~220g) * (triangle pre-pack)	Supermarket OR closest garage nearby		2sl bread + filling			
Fruit salad, canned in juice	Goulburn Valley		700g			

Peanuts – roasted, unsalted peanuts	Cheapest branded		250g			
Tinned steak & vegetables	Harvest		425g			
Discretionary Choices						
Beer (Liquor store)*	VB*		6 x 375mL			
Sparkling white wine (Liquor store)*	Yellow*		750mL			
Whisky (Liquor store)*	Johnny Walker Red Label*		700mL			
Red wine (Liquor store)*	Penfolds Koonungara Hill Shiraz*		750mL			
Butter, original, salted (foil pack)	Western Star		250g			
Muffin, commercial, uniced	Supermarket		$/100g			
Cream-filled biscuit	Arnott's Monte-Carlo		250g			
Chewy Choc Chip Muesli Bar	Uncle Toby's		6x30g (185g)			
Mixed nuts, (incl. peanut), salted	Nobby's		375g			
Supreme Pizza, thin base (1 pizza=0.55kg)*	Pizza Hut*		1 pizza			
Savoury flavoured biscuits	Arnott's BBQ Shapes		175g			
Mint confectionary	Allen's Minties		150g			
Dairy milk chocolate, block	Cadbury		200g			
Soft drink, Cola	Coca Cola		1.25L			
Diet soft drink, Cola	Coca Cola		1.25L			
Chocolate Milk, regular fat	Breaka, Big M,Oak or Paul's		600mL			
Beef Pie, single serve, full pastry*	Independent Bakery*		~250g			
Beef lasagne, frozen	McCain		400g			
Beef hamburger*	McDonald's Big Mac*		1 burger			
Beef Sausages, pre-pack	Supermarket		per kg			
Leg Ham, pre-pack	Don's		250g			
Chips/crisps, original, salted	Smith's or Thins		170g			
Cooked hot potato chips, 1 serve*	Independent Fish & Chip shop*		~110g			
Vanilla Ice cream, regular fat	Nestle Peters Original		2L			
White Sugar	CSR		2kg			
French Dressing, regular fat	Praise		330mL			
Tomato sauce, regular (not ketchup)	Heinz Big Red or Masterfoods		500mL			
Tinned chicken & vegetable soup, ready to eat	Campbell's Country Ladle		505g			
Orange Juice, Australian Grown (Fresh, chilled)	Berri		2L			
White crumbed fish fillet, frozen	Birds Eye		425g			
2 Minute noodles, chicken (cheapest specified brand)	Maggi or Fantastic		70g			
* denotes non-supermarket lines (not all supermarkets stock sandwiches- price at nearest service station/garage)						

Price Collection Protocol

1. Record the *usual price of an item*, i.e. do not collect the sale/special price unless it is the only price available (if so, note in comment column)
2. *Look for the specified brand and specified size for each food item, and record the price*
   - *If the specified brand is not available*: Choose the cheapest brand (non-generic) available in the specified size. Note this brand in the "Your brand" column
   - *If the specified size is not available*: Choose the nearest larger size in the specified brand. If a larger size is not available, choose the nearest smaller size. Note this size in the "Your size" column.
   - *If both the specified brand and specified size are not available*: Choose the cheapest in the nearest larger size of another brand (non-generic). If a larger size is not available, choose the nearest smaller size.
   - *If multiple brands are specified,* record the price of the cheapest one and note brand in the "Your brand" column
   - *If the item is only available in a generic form* (e.g. Home Brand, Coles, Woolworths Select, Black and Gold) choose the *most expensive generic* item in the specified size. If the specified size is not available, choose the nearest larger size. If a larger size is not available, choose the nearest smaller size. Note the generic name in the "Your brand" and the size in the "Your size" columns.
3. *Loose produce*: choose the usual cheapest price per kg of the variety not on special. If the only variety available is on special, record the special price and note in comments column.
4. *Peanuts*: choose the branded packet size closest to 250g. If packaged, roasted, unsalted peanuts are not available, record the price of the loose 'bulk – scoop & weight' roasted, unsalted peanuts per 100g.
5. *Check all data are recorded as above before leaving store.*

For more information contact Prof Amanda Lee, The Australian Prevention Partnership Centre, The Sax Institute
e: Amanda.Lee@saxinstitute.org.au; m: 0412975197
